# Isotope data from amino acids indicate Darwin’s ground sloth was not an herbivore

**DOI:** 10.1038/s41598-021-97996-9

**Published:** 2021-10-07

**Authors:** Julia V. Tejada, John J. Flynn, Ross MacPhee, Tamsin C. O’Connell, Thure E. Cerling, Lizette Bermudez, Carmen Capuñay, Natalie Wallsgrove, Brian N. Popp

**Affiliations:** 1 Institut des Sciences de l’Évolution, UMR 5554, Université de Montpellier, Montpellier Cedex 5, France; 2 American Museum of Natural History, New York, NY USA; 3 Department of Earth and Environmental Sciences, Columbia University, New York, NY USA; 4 Departmento de Paleontología de Vertebrados, Museo de Historia Natural-UNMSM, Lima, Peru; 5 Department of Archaeology, University of Cambridge, Downing Street, Cambridge, CB2 3DZ UK; 6 Department of Geology and Geophysics and Department of Biology, University of Utah, Salt Lake City, USA; 7 Parque Zoológico Huachipa, Lima, Peru; 8 Department of Earth Sciences, University of Hawaii at Manoa, Honolulu, USA

**Keywords:** Biogeochemistry, Palaeontology, Biogeochemistry, Palaeoecology, Stable isotope analysis, Biogeochemistry, Community ecology, Palaeoecology, Stable isotope analysis

## Abstract

Fossil sloths are regarded as obligate herbivores for reasons including peculiarities of their craniodental morphology and that all living sloths feed exclusively on plants. We challenge this view based on isotopic analyses of nitrogen of specific amino acids, which show that Darwin’s ground sloth *Mylodon darwinii* was an opportunistic omnivore. This direct evidence of omnivory in an ancient sloth requires reevaluation of the ecological structure of South American Cenozoic mammalian communities, as sloths represented a major component of these ecosystems across the past 34 Myr. Furthermore, by analyzing modern mammals with known diets, we provide a basis for reliable interpretation of nitrogen isotopes of amino acids of fossils. We argue that a widely used equation to determine trophic position is unnecessary, and that the relative isotopic values of the amino acids glutamate and phenylalanine alone permit reliable reconstructions of trophic positions of extant and extinct mammals.

## Introduction

Home to more than half of Earth’s land biota, South America contains the highest diversity of extant terrestrial mammals of any continent^1^. Little is known, however, about the ecological structure of mammalian communities before the megafaunal extinctions of the last Pleistocene ice age, when more than 80% of mammals above 40 kg became extinct^2^. Characterizing the paleoecology of South American (SA) fossil mammals is particularly challenging, because those communities bear little resemblance to modern or fossil guilds from other continents due to marked taxonomic and phylogenetic disparities and unusually large numbers of entirely extinct clades^3^. Another aspect of this perceived uniqueness is the depauperate abundance and diversity of mammalian carnivores throughout the Cenozoic. Lack of placental mammalian carnivores is particularly puzzling for the Pleistocene because all large marsupials (e.g., borhyaenoids) and non-mammalian predators such as terrestrial crocodiles (i.e., sebecids) and large flightless “terror birds” (i.e., phorusrhacids), which likely maintained terrestrial food web energy transfer balances in the Tertiary, were already extinct by this epoch. Allometric functions relating population densities to body mass indicate that Pleistocene SA mammalian communities were ecologically imbalanced because the herbivore biomass markedly surpassed the energetic requirements of carnivores^4^. It has been further suggested that Pleistocene ecosystems could not have supported the plant biomass demands of all the presumed large herbivores, which implies that secondary consumers disguised as herbivores must exist in the fossil record but have not been recognized as such^4^. Because of their high taxonomic diversity and simple yet potentially functionally versatile dentitions, fossil sloths (Xenarthra, Folivora) have been identified as the most likely candidates to have occupied scavenger niches^4,5^. All modern sloth species, however, are obligate tropical arboreal herbivores, with extreme feeding specializations involving the exploitation of food of low nutritional quality, with the extra physiological challenges entailed by the poor digestibility of plant allelochemicals^6^. The traditional consensus that fossil sloths were also obligate herbivores like their modern relatives is based mostly on craniodental morphology analyses and a presumption of shared, phylogenetically conserved dietary adaptation across the group. Dental structures and jaw biomechanics of sloths, however, do not preclude the ingestion of foods that require little or no preparation, as in certain kinds of scavenging (e.g., ingestion of animal soft tissues where minimal or no chewing is involved). Furthermore, their extremely high diversity and incredibly broad range of body sizes, geographic distributions, and inferred habitats, including even a marine sloth^7^, suggest that some of the hundreds of known species of fossil sloths may have been much more versatile ecologically than traditionally thought. Sloths (together with other xenarthrans, such as anteaters, armadillos, and extinct close relatives) comprised a substantial proportion of South American mammalian diversity until as recently as 10,000 years ago^8^. Reliably determining the trophic relationships of xenarthrans is thus crucial for understanding the evolution of Neotropical mammalian biodiversity, as well as the interplay between resource availability, ecological functions among xenarthrans, and competition within and across mammalian clades in South America over the Cenozoic.

### Trophic inferences using stable isotopic composition

Isotopic analyses are now widely applied in ecological and paleoecological studies. These techniques rely on two underlying premises. First, that isotopic values of any given chemical element differ across diverse reservoirs in the biosphere, yielding isotopically varied foods and waters. Second, because an organism’s body tissues are synthesized from the food and water they ingest, they are isotopically linked to those sources in a predictable way^9^. Stable isotope analyses therefore offer a rare route to directly document the consumed diet itself, rather than relying on indirect inferences based on morphological proxies, representing a particularly useful and potentially more reliable method to be applied to fossils. Traditionally, nitrogen stable isotope analyses (δ^15^N) of bulk tissue are used to evaluate trophic levels due to an empirically documented increase of 3–5‰ in δ^15^N values per trophic level change^10,11^. However, substantial variation in δ^15^N values has also been observed within trophic guilds^11,12^, indicating that the use of δ^15^N values alone can be a misleading proxy to identify trophic levels. Indeed, interpretation of bulk tissue δ^15^N values often fails to acknowledge variations in baseline δ^15^N values within an ecosystem^13^, which can greatly differ depending on the isotopically different types of inorganic nitrogen sources available for the primary producers (e.g., N_2_, NH_3_, NO^−^_3_^14^), the way nitrogen was obtained (direct uptake of nitrogen from soil or through symbiotic microbes), and where nitrogen was assimilated by the plant (e.g., root, shoot).

In contrast, amino acid compound-specific isotope analysis (AACSIA), a more recent technique that measures and evaluates the δ^15^N values of specific amino acids, overcomes the issue of unknown or variable baseline δ^15^N values because it records both baseline (i.e., that for the overall primary producer community) and trophic information in the same organism^15–18^. Indeed, studies have shown that certain amino acids (AAs) experience little change in their δ^15^N values from ingested diet to consumer body tissues, whereas others differ significantly^15^. The explanation behind this observation relies on whether or not the metabolic pathways of amino acids are engaged in transamination or deamination reactions (involving the breakdown of C-N bonds) and the extent to which amino acids exchange their amino-nitrogen with the overall metabolic pool of nitrogen^19^. Amino acids that readily exchange their amino-nitrogen, thereby experiencing large isotopic fractionations, have been termed “trophic” amino acids, whereas those implicated in metabolic processes that involve little or no breakdown or formation of C-N bonds, and thus are similar to baseline δ^15^N values, are known as “source” amino acids^17,19^. The higher the trophic level of an organism, the more metabolic cycles their amino-nitrogen will be involved in, and therefore the difference between the δ^15^N values of trophic versus source amino acids will progressively increase. Among “trophic” AAs, glutamic acid (Glx, analyzed as glutamate) usually experiences a large increase in its δ^15^N values with each trophic level transfer^20^, as its central role in nitrogen metabolism of heterotrophs involves transamination reactions^19,21^. In contrast, among source amino acids, phenylalanine (Phe) has been observed to experience minimal increase in its δ^15^N values per each trophic level, as its main initial metabolic step, the formation of tyrosine, does not involve breaking C–N bonds^20–22^. Constancy of these distinct patterns between Glx and Phe across organisms makes them the two canonical “trophic” and “source” amino acids^15–17,23^. Indeed, the most popular equation ^20^ to calculate trophic positions (hereafter referred to as “TP Eq”) uses the δ^15^N values of those two amino acids, although a few studies have instead proposed a combination of multiple trophic and source amino acids to calculate trophic levels^14,24,25^.

Although widely applied in archaeological, anthropological, and paleontological research (e.g., to evaluate dietary practices of ancient humans and other hominoid species (e.g.,^23,26^), application of the AACSIA TP Eq is not without caveats, as this equation relies on constants (β value and trophic discrimination factor [TDF]) that have not been thoroughly evaluated in terrestrial ecosystems. Indeed, the equation relies on the assumption that the β value (isotopic differences between glutamate and phenylalanine [“big delta” Δ] in primary producers) and the TDF (^15^N-enrichment of glutamate relative to phenylalanine per trophic level change [Δ_Glx_ − Δ_Phe_]) are both constant across all organisms within an ecosystem. For terrestrial ecosystems, β values of − 8.4‰ and − 0.4‰ are considered representative of C_3_ and C_4_ plant-dominated ecosystems, respectively^20,27^; however, a recent meta-analysis of β values, demonstrated considerably more variation than currently acknowledged^28^. On the other hand, a standard TDF value of 7.6‰ has been used to calculate trophic positions of all consumers, whether terrestrial or aquatic, modern or extinct, vertebrate or invertebrate, yet its assumption of constancy has only been tested on marine invertebrates, fishes, birds, insects, and heterotrophic bacteria, where it is also known to vary^16,20,29–31^. The only prior controlled experimental study we are aware of on a terrestrial mammal, *Bos taurus*^32^, showed different β (− 4.5‰) and TDF (4‰) values than those used in the TP Eq, suggesting that these “universal” values cannot be applied uniformly across food webs and ecosystems.

For these reasons, we posit that the TP Eq cannot be used to confidently calculate the trophic position of mammals (also see^33^). We argue instead that this equation is not necessary in any case, and that the δ^15^N values of glutamate (δ^15^N_Glx_) and phenylalanine (δ^15^N_Phe_) alone permit reliable determinations of trophic positions of mammals. To document this, we first tested the predictive power of this relationship in modern mammalian species under different, known feeding regimes or diets (in both controlled and wild environments), to provide a basis for reliable interpretation of δ^15^N AACSIA of fossil samples. We then used this proxy to test the hypothesis of carnivory and consumption of proteins of animal origin in the targeted fossil sloths, *Mylodon darwinii* and *Nothrotheriops shastensis*.

## Results

### δ^15^N of Glx and Phe

The seven xenarthran species included in this study (sloths [extant and extinct] and anteaters) display substantial intra- and inter-specific variation in their δ^15^N_Phe_ values (> 15‰ across individuals, Table 1, Table S1), with the spectrum of δ^15^N_Phe_ variation being bracketed by the two fossil sloth species, *Mylodon darwinii* (median δ^15^N_Phe_ = 2.5‰) and *Nothrotheriops shastensis* (δ^15^N_Phe_ = 17.7‰). The greatest intraspecific δ^15^N_Phe_ variation (8.4‰ span) is observed in *Choloepus* (fed an omnivorous diet in the zoo, SI), with hair keratin δ^15^N_Phe_ values ranging from 3.2 to 11.6‰. Intraspecific variation in δ^15^N_Glx_ values is smaller than that for δ^15^N_Phe_, with *Choloepus* and *Mylodon* showing the greatest variation among individuals (2.5‰). Inter-specific variation in δ^15^N_Glx_ spans 14‰, with the extreme values also bracketed by the fossil sloths *Mylodon* (median δ^15^N_Glx_ = 5.6‰) and *Nothrotheriops* (δ^15^N_Glx_ = 19.6‰).

**Table 1:** AA and bulk tissue δ15N values (‰ vs. AIR) for modern (zoo) and fossil (†) sloth species (n = 3 for all species except *Nothrotheriops*).

Sample	δ^15^N														∆ Glx-Phe
	Ala	Gly	Thr	Ser	Val	Leu	Iso	Pro	Asx	Glx	Phe	Tyr	Lys	Bulk	
*Bradypus variegatus*	12	11.9	0.3	14	14.7	11.5	14.8	15.2	12	14.6	15.3	11.9	9.7	10.5	− 0.7
	12.3	10.1	− 1	13	13.3	11.9	14.2	15.8	11.6	14.5	14.1	11.6	11.1	10.6	0.4
	9.7	11.3	0.7	13	12.8	11.7	10.4	14.9	11.3	14.1	12.9	7.8	6.6	10.8	1.2
Span	2.6	1.8	1.7	1	1.9	0.4	4.4	0.9	0.7	0.5	2.4	4.1	4.5		
Median	12	11.3	0.3	13	13.3	11.7	14.2	15.2	11.6	14.5	14.1	11.6	9.7		0.4
*Choloepus hoffmanii*	7.8	5.1	− 7.6	9.3	9	8.0	9.7	11.1	8.3	11.2	3.2	3.0	2.4	7.1	7.9
	9.2	4.3	− 5.5	8.9	9.3	8.1	10.8	11.5	8.6	11.5	11.6	3.8	2.3	7.6	− 0.1
	11.5	6.7	− 5.3	10	8.5	10.6	12.4	13.5	12.1	13.7	8.1	6.1	5.7	7.6	5.5
Span	3.7	2.2	2.3	1.1	0.8	2.6	2.7	2.4	3.8	2.5	8.4	3.1	3.4		
Median	9.2	5.1	− 5.5	9.3	9	8.1	10.8	11.5	8.6	11.5	8.1	3.8	2.4		5.5
†*Mylodon darwinii*	1.7	− 0.4	− 7.2	4.9	4.2	1.1	3.3	3.8	3.1	4.6	2	− 4	− 2.5	2	2.6
	2.9	1.5	− 5.7	5.5	7.6	3.3	5.9	5.9	4.5	5.6	2.5	− 3.4	− 3.6	3.4	3.1
	6.6	2.2	− 7.8	5.0	8.1	4.5	6.7	6.9	5.9	7.1	4.4	− 1.3	− 1.1	3.7	2.7
Span	4.9	2.6	2.1	0.6	3.9	3.4	3.4	3.1	2.8	2.5	2.4	2.7	2.5		
Median	2.9	1.5	− 7.2	5.0	7.6	3.3	5.9	5.9	4.5	5.6	2.5	− 3.4	− 2.5		2.7
†*Nothrotheriops shastensis*	15.6	13.3	4.5	17	16.7	15.9	17.9	18.4	15.8	19.6	17.7	12.8	9.8	16.4	1.9

∆Glx-Phe is the offset between δ^15^N_Glx_ and δ^15^N_Phe_ values. Notice that the high ∆Glx-Phe in the two-toed sloth *Choloepus* is because it is fed a mixed animal-plant omnivorous diet at the zoo.

The δ^15^N values of bulk dietary samples for zoo specimens are bracketed by the results for the two living sloth species, the mixed plant/animal diet of *Choloepus* (δ^15^N_Phe_ = 4.5‰, δ^15^N_Glx_ = 5.3‰) and the rubber plant *Ficus elastica*, *Bradypus*’s monospecific diet (δ^15^N_Phe_ = 18.6‰, δ^15^N_Glx_ = 11‰; Table S2). The offset between the δ^15^N values of glutamic acid and phenylalanine (β value) for *Ficus elastica* is − 7.6‰ (Table S3). Mixed component (“omnivorous”) zoo diets show similar offsets in their δ^15^N_Glx_-δ^15^N_Phe_ values (Δ), ranging from − 1‰ for the diet of the pygmy anteater *Cyclopes* to 0.8‰ for that of *Choloepus*. The dietary Δδ^15^N_Glx_ − δ^15^N_Phe_ result for *Myrmecophaga* and *Tamandua* (both species fed the same diet at the zoo) was − 0.4‰ (Table S3).

**Figure 1:**
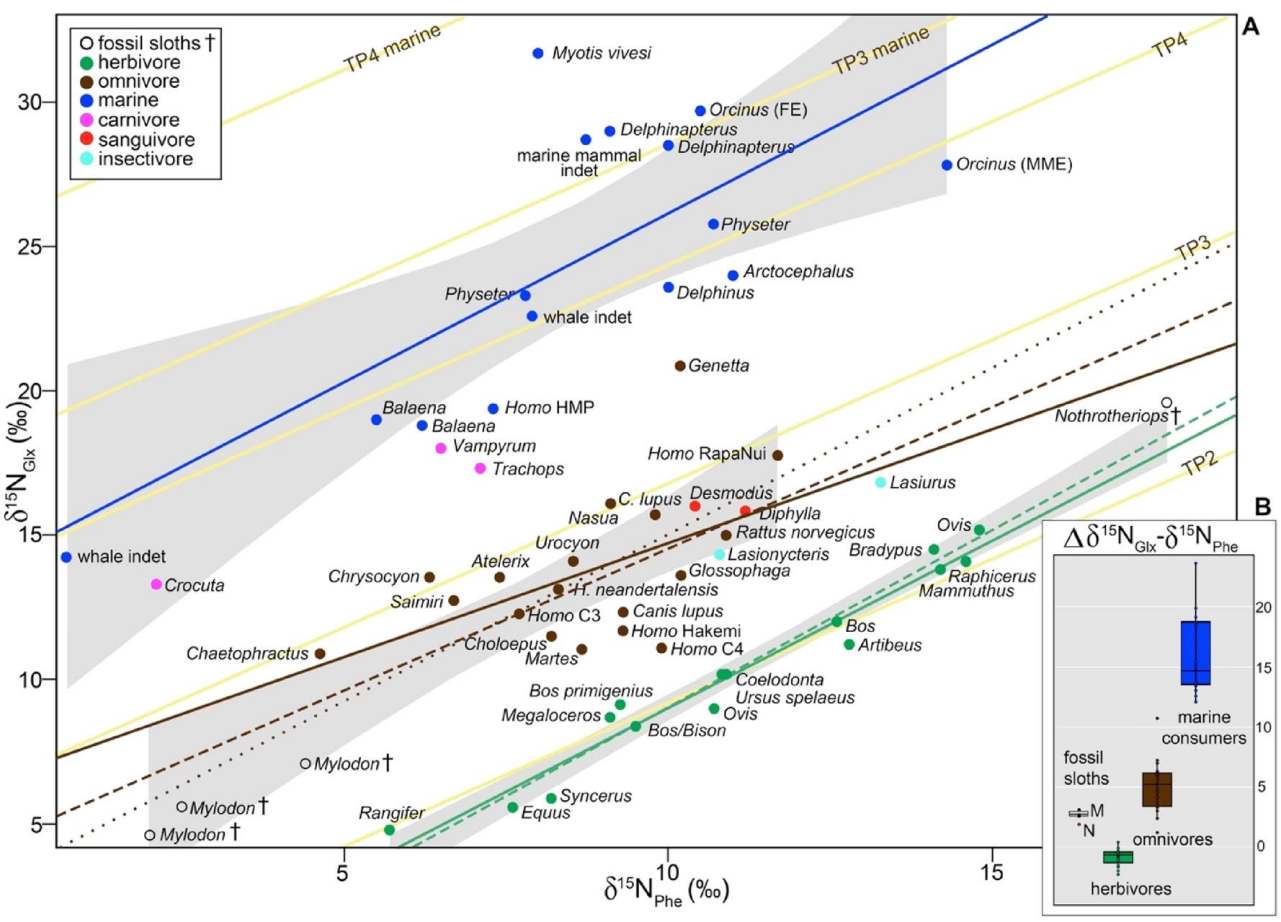
Scatter plot of δ^15^N_Glx_ and δ^15^N_Phe_ (**A**) and ∆δ^15^N_Glx_ − δ^15^N_Phe_ (**B**) for mammals, separated by feeding ecology categories. (**A**) Regression lines plotted for: modern herbivores (n = 15, solid green line, R^2^ = 0.96, *p* < 0.001); modern herbivores + fossil *Nothrotheriops* (dashed green line, R^2^ = 0.96, *p* < 0.001); modern wild omnivores (i.e., excluding zoo anteaters, n = 18) (solid brown line, R^2^ = 0.28, *p* = 0.03), modern omnivores + both fossil sloth species (dashed brown line, R^2^ = 0.68, *p* < 0.001), modern omnivores + fossil *Mylodon* (while excluding the two outliers of this regression *Nothrotheriops* and *Genetta* [determined by robust regression and diagnostic analyses], dotted brown line, R^2^ = 0.71, *p* < 0.001). Grey shading is the standard error, including *Mylodon* with omnivores and *Nothrotheriops* with herbivores. Yellow lines represent theoretical trophic levels from the TP equations by^20^. Notice that except for primary consumers (TP = 2), the theoretical trophic levels obtained from those equations do not match the known feeding ecologies for mammals in higher trophic levels. Data used for this figure comes from this study and a thorough literature compilation (Table S6). (**B**) M = *Mylodon*, N = *Nothrotheriops*.

Because baseline δ^15^N_Phe_ and δ^15^N_Glx_ values can vary across ecosystems, what is actually informative for trophic position inferences is how δ^15^N_Glx_ and δ^15^N_Phe_ values compare to each other in the animal under study (e.g., the absolute difference [“big delta” Δ] or as reflected in linear regression models, Fig. 1). Significant differences in the Δδ^15^N_Glx_ − δ^15^N_Phe_ are observed among species in distinct dietary categories (*p* < 0.001, Fig. 1, Table 2, Table S4, S5). The median Δδ^15^N_Glx_ − δ^15^N_Phe_ of herbivores is significantly lower (− 0.7‰, n = 15) than that of omnivores (4.6‰, n = 21; including zoo animals fed mixed-diets [plant/animal], t(21.9) = − 10.2, *p* < 0.001) and marine consumers (15.5‰, n = 17, *p* < 0.001) (Table 2, Table S4, S5). Among herbivores, the range of variation in Δδ^15^N_Glx_ − δ^15^N_Phe_ (2.7‰) is bracketed by two artiodactyls, the buffalo *Syncerus caffer* (− 2.3‰) and sheep *Ovis aries* (0.4‰, Table S6). The range of variation in Δδ^15^N_Glx_ − δ^15^N_Phe_ among omnivores is large (9.5‰), bracketed by humans with high plant content in their diets (1.2‰; C_4_ consumers *sensu*^34^) and the common genet *Genetta genetta* (10.7‰). The range of variation in Δδ^15^N_Glx_ − δ^15^N_Phe_ in marine consumers also is large (11.6‰), ranging from 12.1‰ in a population of *Homo sapiens* from South Africa to 23.7‰ in the fish-eating bat *Myotis vivesi*. The ∆δ^15^N_Glx_ − δ^15^N_Phe_ values in marine consumers are significantly higher (*p*-value < 0.001) than for any terrestrial feeding guild (i.e., herbivores and omnivores). Data for terrestrial carnivores is limited to three species with ∆δ^15^N_Glx_ − δ^15^N_Phe_ values lower than for all marine consumers: the hyena *Crocuta crocuta* (∆δ^15^N_Glx_ − δ^15^N_Phe_ = 11.2‰), the fringe-lipped bat *Trachops cirrhosus* (∆δ^15^N_Glx_ − δ^15^N_Phe_ = 10.2‰), and the spectral bat *Vampyrum spectrum* (∆δ^15^N_Glx_ − δ^15^N_Phe_ = 11.5‰). Fossil sloths were not placed in any dietary category a priori. The Δδ^15^N_Glx_ − δ^15^N_Phe_ values for *Mylodon* overlap those of taxa within the omnivore category (median = 2.7‰) while *Nothrotheriops* best fit values in the herbivore category (median = 1.9‰, Fig. 1, Table S5).

**Table 2:** AA and bulk δ^15^N values (‰ vs. AIR) for the eight modern omnivorous mammalian species analyzed.

Sample	δ^15^N														∆ Glx-Phe
	Thr	Gly	Lys	Ser	Ala	Asx	Iso	Leu	Pro	Val	Tyr	Glx	Phe	Bulk	
*Chrysocyon jubatus*	− 13.1	5.7	2.1	9.2	10.3	10	10.4	10.4	10.6	10.8	3.6	13.5	6.3	7.7	7.2
*Chaetophractus vellerosus*	− 13.3	5.5	4	8.2	8.9	7.7	9.6	8.6	11.3	10.5	3.2	10.9	4.6	6.5	6.3
*Saimiri boliviensis*	− 11.2	4.5	1.6	8.5	11.1	12.3	10.6	9.9	15	11.3	−	12.7	6.7	7.2	6
*Atelerix frontalis*	− 13	8.6	5.7	9.8	11.9	14.2	11.6	10.4	16	11.8	− 0.5	13.5	7.4	9.4	6.1
*Genetta genetta*	− 10.1	10.6	10.4	15.7	19.7	15.9	18.2	18.5	22.9	19.3	12.9	20.9	10.2	14.6	10.7
*Urocyon cinereoargentus*	− 10.7	5.6	1.8	8.4	12.3	10.1	10.4	11.1	14.3	12.7	5.9	14.1	8.5	8.5	5.6
*Nasua nasua*	− 13	5.9	5.2	10.8	14.3	12.3	15.2	14.1	18.1	15.6	3.9	16.1	9.1	9.8	7
*Martes americana*	− 19.5	2.6	− 0.7	6.1	9.6	7.3	10.2	8.5	9.5	10	2.3	11.1	8.7	6.6	2.4

∆Glx-Phe as in Table 1.

Ordinary least square (OLS) analyses of δ^15^N_Glx_ and δ^15^N_Phe_ for 59 mammalian species yielded strong correlations between these two AAs for herbivores (R^2^ = 0.96, *p*-value < 0.001, SE = 0.7 on 13 degrees of freedom [df]), marine consumers (R^2^ = 0.52, *p*-value = 0.002, SE = 3.6 on 13 df), and wild omnivores (R^2^ = 0.28, *p*-value = 0.03, SE = 2.3 on 16 df) (Fig. 1, Table 2, Table S5). The ∆δ^15^N_Glx_ − δ^15^N_Phe_ values in modern mammals increase per trophic level change (Fig. 1, Table S5). Values for herbivores and omnivores are notably different and do not overlap (Fig. 1, Table S5), when excluding the zoo anteaters (removed because of their artificial dietary components differing markedly from their specialized insectivore diet in the wild, see SI). Analysis of covariance was used to assess the effect of dietary classification (herbivores, omnivores, and marine consumers) on the δ^15^N_Glx_ and δ^15^N_Phe_ interaction (i.e., statistical differences in slopes and intercepts among regression lines, with δ^15^N_Glx_ modelled as the dependent variable). Our results show that there is a significant effect of diet on δ^15^N_Phe_, but no significant interaction (i.e., the slopes are not significantly different; F(1, 43) = 0.36, *p* = 0.78; Table S4). In contrast, diet has a significant effect on δ^15^N_Glx_, observed as differences in intercepts among the regression lines (F(3, 46) = 116.5, *p* < 0.01; Table S4).

Additional ordinary least square (OLS) analyses and robust regressions were carried out classifying fossil sloths either as herbivores or as omnivores, to evaluate the best dietary category fit for these species relative to known dietary correlations in extant taxa (Fig. 1). Fossil sloths were not placed a priori in any dietary category in the initial analyses that included only living taxa. When the OLS analysis was run with both fossil sloths classified as herbivores, the correlation between δ^15^N_Glx_ and δ^15^N_Phe_ remained strong (*p* < 0.001), but R^2^ decreased from 0.96 to 0.86. When only *Nothrotheriops* is considered as an herbivore, however, the correlation between δ^15^N_Glx_ and δ^15^N_Phe_ for herbivores is as strong (R^2^ = 0.96, *p* < 0.001) as when only modern taxa are analyzed (Fig. 1, Table S5). When both fossil sloths were classified as omnivores, the correlation between δ^15^N_Glx_ and δ^15^N_Phe_ for this dietary category is strong (R^2^ = 0.7, *p* < 0.001). However, results from robust regression and diagnostic analyses of fossil and extant taxa together identified *Nothrotheriops* (along with *Genetta*) as influential outliers for the omnivorous correlation (Cook’s D > 4/n). The exclusion of these two outliers, and inclusion of *Mylodon* as an omnivore, greatly improved the strength of the omnivore correlation (R^2^ = 0.71, *p* < 0.001, Fig. 1, Table S5).

## Discussion

Our results document that we can accurately reconstruct the trophic position of mammal species from δ^15^N_Glx_ and δ^15^N_Phe_ values alone (both the ∆ value and correlations between the two amino acids), which are therefore preferable to relying on an equation with poorly constrained constants introducing uncertainties. Indeed, the TP Eq often failed to place captive and wild mammalian omnivores in their correct trophic positions (as was also observed in other mammalian^33^ and non-mammalian organisms^35,36^, Table S6). Misidentification of many mammalian omnivores using the TP Eq might be associated with the consumption of dietary items of mixed origin (C_3_, C_4_, marine, or a combination of those), which introduces uncertainties that are not accounted for when using single constant β and TDF factors (see discussion of predictive power of the TP Eq for mammals in SI). Low ∆δ^15^N_Glx_ − δ^15^N_Phe_ values for the captive anteaters (− 2.2 and − 0.3 for *Tamandua* and *Cyclopes* respectively) are likely due to the fact that these individuals are fed diets containing protein supplements with higher δ^15^N_Phe_ values than expected for secondary consumers, consequently blurring the ∆δ^15^N_Glx_ − δ^15^N_Phe_ values naturally observed in other species in that trophic level. The weaker correlation between δ^15^N_Glx_ and δ^15^N_Phe_ for wild omnivores compared to herbivores or marine consumers is likely due to the fact that this feeding category includes mammals incorporating widely different proportions of distinct source foods (e.g., fruits/plants, invertebrates, and vertebrates) in their diets.

**Figure 2:**
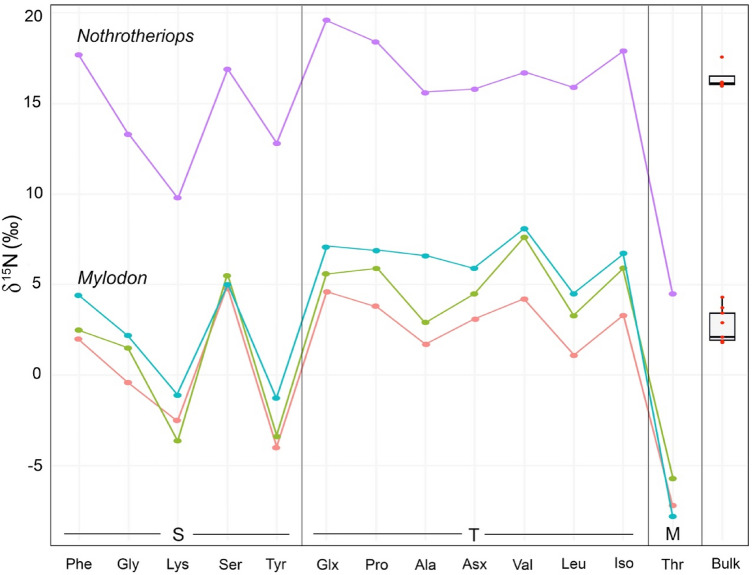
Nitrogen isotope values of individual amino acids and bulk hair for the two fossil sloth species *Mylodon darwinii* and *Nothrotheriops shastensis*. S = source AAs, T = trophic AAs, M = metabolic AA. Variation in bulk δ15N reflect variations in hair sections as described in main text (also see Fig S1).

Based on the OLS analysis and their ∆δ^15^N_Glx_ − δ^15^N_Phe_ values (Fig. 1), the two fossil sloths, *Mylodon* and *Nothrotheriops,* occupy distinct trophic positions, but in contrast to what might be interpreted from bulk δ^15^N values (Fig. 2, Table S7), *Mylodon* is in a higher trophic position than *Nothrotheriops*. Indeed, *Mylodon* better fits the omnivore linear regression and its ∆δ^15^N_Glx_ − δ^15^N_Phe_ values (2.6 to 3.1) match those of modern omnivores, such as the American marten. In contrast, although the ∆δ^15^N_Glx_ − δ^15^N_Phe_ value obtained for the fossil sloth *Nothrotheriops* (1.9) fit that of some low-meat consuming omnivores (e.g., *Homo sapiens* consuming C4 plant resources), it was found as an outlier in the omnivore regression and thus is conservatively interpreted as an herbivore (Table S5). There is no evidence for consumption of items of marine origin in either sloth species, which is not surprising for *Nothrotheriops*, mostly known from continental interior desert habitats in the southwestern U.S., but rules out potential dietary incorporation of available marine resources for *Mylodon*, which lived near coastal areas. The remarkably high AA and bulk δ^15^N values of *Nothrotheriops* (the δ^15^N_Phe_ value is higher than any other mammal analyzed, terrestrial or marine) is likely due to the arid conditions in which this animal lived, as plant δ^15^N values increase with decreasing water availability^37,38^ and the taxonomic composition of the plants identified in its diet (from dung) consistently correspond to desert taxa still present in the area^39,40^.

The offset between δ^15^N_Glx_ and δ^15^N_Phe_ values (i.e., β value) is significantly different in plants performing C_3_ (~ − 8.4‰) versus C_4_ (~ 0.4‰) photosynthesis^20^, although substantial variation also exists among C_3_ plants (e.g., ranging from − 2.6‰ in *Urtica doica* to − 12.2‰ in *Sambucus nigra*) depending on the degree of vascularization^28^ and amount of lignin produced by the plant species^27^. Mammalian herbivores, whether feeding on C_3_ or C_4_ resources, however, do not differ significantly in ∆δ^15^N_Glx_ − δ^15^N_Phe_ values (*p*-value = 0.77, SI Dataset). Thus, the almost 1:1 correlation between these two amino acids in terrestrial herbivores is independent of the type of plant consumed (Fig. 1), and therefore also independent of ecosystem. This observation gives an undeniable power to the δ^15^N_Glx_ − δ^15^N_Phe_ correlation to predict herbivory. This also implies that C_3_- and C_4_-consuming herbivores have disparate patterns of amino acid δ^15^N discrimination, with significantly large ^15^N enrichment in the Glx of C_3_ herbivores versus minimal enrichment in C_4_ herbivores. Data on controlled-diet zoo mammals feeding on a single plant species (e.g., the three-toed sloth *Bradypus* feeding on *Ficus elastica* [this study] and the cow *Bos taurus* feeding on *Lolium perenne*^32^) and data on wild mammals feeding on C_3_ or C_4_ plants (SI Dataset) both corroborate this observation.

Assessing the results of all analyses together (∆δ^15^N_Glx_ − δ^15^N_Phe_ and linear relationships between δ^15^N_Glx_ and δ^15^N_Phe_ in extant mammals) suggests that while the Shasta ground sloth *Nothrotheriops shastensis* was likely an obligate herbivore, Darwin’s sloth *Mylodon darwinii* was not, instead occupying a higher trophic position. AA δ^15^N values indicates that the feeding ecology of the fossil sloth *Mylodon darwinii* best fits within the mixed-feeder/omnivore dietary category, with a degree of meat-eating omnivory comparable to that of the extant American marten. Further, the strength of the correlation between δ^15^N_Glx_ − δ^15^N_Phe_ for modern herbivores, regardless of its ecosystem (specimens analyzed live in a wide array of ecosystems) or the type of vegetation consumed (C_3_/C_4_, woody/non-woody plants) makes it extremely unlikely for *Mylodon* to have been an obligate herbivore.

### Comparison of δ^15^N AACSIA results to interpretations from other proxies

Macroscopic analysis and DNA sequences extracted from *Nothrotheriops shastensis* coprolites (from Rampart and Gypsum Caves in the US^39,41^), revealed a variety of plant species but no evidence for a non-herbivorous diet. *Mylodon darwinii* dung has been subject to ancient DNA studies aimed to explore evolutionary relationships of this species^42,43^, but only one study attempted to characterize its diet from dung^44^. This macro- and micromorphological study of dung content found *Mylodon*’s diet to be composed mostly of grasses and sedges, concluding that it most likely was a grazer^44^, in contrast to morphological interpretations^45^. The absence of hardly digestible material (e.g., bones) in any *Mylodon*’s coprolites shows that this species could not have been a strict carnivore (or a hyena-type scavenger) but does not exclude an opportunistic omnivore behavior because other animal protein-rich dietary items (e.g., meat, eggs) are rapidly assimilated after ingestion. In this case, fecal components will be biased towards plant dietary items because cellulose is difficult to digest. Indeed, macro- and micromorphological analyses of feces do not allow precise identification of an animal’s full dietary composition, as has been exhaustively demonstrated in archaeological studies of human coprolites^46^.

Anatomical studies on modern herbivores, on the other hand, showed a strong correlation of dental occlusal surface area (OSA) with efficiency and capacity of food processing, digestive physiology, and food quality^47^. Hindgut fermenting grazers, such as horses, have greater relative OSAs than do cows (ruminant foregut fermenters); by being more efficient at orally reducing the particle size of the ingested food, horses compensate for shorter digesta retention times than ruminants^48^. Compared to modern herbivores, fossil sloths (with the sole exception of *Megatherium americanum*) had smaller occlusal areas than expected for their body masses^49^, indicating that they had a lower capacity for food processing than do extant herbivores of similar sizes.

Of all fossil sloth species analyzed, mylodontids (the clade including *Mylodon darwinii*) had the lowest relative OSA values, reflecting poor oral food processing efficiency. These results were interpreted as likely due to lower energetic requirements of fossil sloths, suggesting in turn that fossil sloths might have required lower food intake than other mammals of similar sizes^49^. Although low basal metabolic rates might partially account for these seemingly incongruent digestive physiology inferences, there is no compelling evidence for unusually low metabolic rates in fossil sloth species. McNab^50^ proposed that *Mylodon* and *Nothrotheriops* would have had basal metabolic rates of 50% of that expected from their body mass. Those estimations are not convincing, however, because they were based on the length of hair strands and fur thickness, while energy transfer along hairs also depends on other traits such as hair diameter and number and arrangement of hair in the fur^51^. Even assuming those estimations were roughly correct, *Mylodon* is thought to have faced ambient temperatures well below its thermoneutrality^50^, and therefore it would have needed to increase its metabolism to maintain a constant body temperature in cooler conditions. Furthermore, a fact considered crucial in explaining the low metabolic rates in modern xenarthrans is their food preferences, including plant items with extremely low energy content and allelochemical defenses against herbivory^50^.

That fossil sloths were foregut fermenters is highly likely because extant sloths, phylogenetically bracketing almost the entire diversity of fossil sloths, are foregut fermenters^6^. Assuming that their digestive efficiencies were at least comparable to that of modern ruminants, however, poses a physiological and anatomical problem because Plio-Pleistocene sloths reached larger body masses than the largest extant foregut fermenter, the hippo (considered to have attained the upper body size limit for non-ruminant foregut fermenters^52^). Foregut fermenters tend to have smaller large intestines (namely, the colon descendens) than hindgut fermenters, which results in reduced colonic water reabsorption^52^. The foregut of *Mylodon* would have represented ~ 20%− 24% of its body weight (calculation based on the reticulorumen equation of^52^, assuming a body mass of 1500–2000 kg, SI) which is comparable to that observed in modern hippos. However, high water loss resulting from a reduced caecum and colon in the hippos is hypothetically overcome by their amphibious lifestyles^52^. An amphibious lifestyle for *Mylodon*, however, has never been proposed, nor would one be consistent with habitats in which they are known to have lived in. For other mylodontids, such as the giant ground sloth *Lestodon* (estimated weight > 4 tons), the issue of colonic water reabsorption is even more extreme because their foregut would have represented almost 40% of their total weight (even assuming that these were browsers, and not grazers as suggested by craniodental morphology studies^45^; calculations the same as above]).

An alternative explanation for these results on fossil sloths, and *Mylodon* in particular, would be ingestion of a higher quality of food. Indeed, if *Mylodon* was an opportunistic scavenger and incorporated, even if only sporadically, meat or other animal dietary elements, then its relatively small oral food processing capacity would be more congruent with the apparent physiological constraints imposed by its body mass and foregut fermentation strategy. The energetic requirements of *Mylodon*, with its need for higher metabolic rates due to air temperatures below its thermoneutrality, also are more consistent with omnivory than obligate herbivory. Our δ^15^N AACSIA results support this interpretation, as they clearly indicate that *Mylodon* was not an obligate herbivore, but instead was an omnivore showing consumption of animal proteins in proportions comparable to the American marten and other mixed feeders.

## Conclusions and implications

Increased sampling and a new analytical framework for AACSIA data document the high predictive power of relationships between δ^15^N_Glx_ and δ^15^N_Phe_ values in modern mammals with known diets, and from distinct ecosystems, digestive physiologies, and phylogenetic relationships. These relationships indicate that analyses of δ^15^N_Glx_ and δ^15^N_Phe_ alone are preferable to inferences of trophic position using an equation (TP Eq) with inaccurate or poorly documented assumptions, and permit reliable reconstruction of diets in fossil taxa from terrestrial ecosystems. More δ^15^N_Glx_ and δ^15^N_Phe_ data on modern secondary consumers may permit differentiation of insect-eating from meat-eating omnivores, as well as from terrestrial hypercarnivores, by teasing apart patterns of interspecific variation in δ^15^N_Glx_ and δ^15^N_Phe_ relative to particular food sources for omnivores.

The newly established relationships between dietary category and δ^15^N_Glx_ and δ^15^N_Phe_, enable us to conclude that while *Nothrotheriops* was likely an obligate herbivore, *Mylodon* was not. Our data, from direct isotopic evidence of the food consumed, clearly indicate that *Mylodon* was not an exclusive herbivore, as commonly presumed, but rather that its feeding behavior better fits that of an omnivore, consuming plant material but sometimes also incorporating items of animal origin in its diet. Although the craniodental and skeletal morphology of sloths does not support them being active predators, an opportunistic scavenging behavior for *Mylodon* would be in agreement with our results obtained via AACSIA. Our conclusions, from direct isotopic evidence of the food consumed, are also supported by studies on masticatory muscle structure reconstructions, biomechanics, and morphogeometry for *Mylodon*, indicating that it would have been inefficient at processing food and at a disadvantage (in terms of resource competition) relative to other mammalian herbivores^49^.

These results, providing the first direct evidence of omnivory in an ancient sloth species, demands reevaluation of the entire ecological structure of mammalian communities in Cenozoic South America, as sloths represented a major component of these ecosystems across the past 34 million years. For instance, in the well-studied Pleistocene Lujanian fauna in Argentina, xenarthrans would have represented almost half of the mammalian herbivore guild, with sloths alone representing almost 20%^53^. Furthermore, as large herbivores greatly impact the vegetation structure, soil moisture, and the carbon cycle of an ecosystem^54^, removal of some fossil ground sloth taxa from the continental herbivore guild would change previous estimations of the net primary production required to sustain a given number of megaherbivores, as well as the type of vegetation dominating the biome floor (e.g., grass-dominated versus moss/shrub-dominated^55^). This would be the case in particular if, in addition to *Mylodon*, other fossil sloth species also had more versatile feeding behaviors than traditionally thought and would help explain the perplexing and long-assumed paucity of mammalian secondary consumers during the Cenozoic in South America.

## Materials and methods

We analyzed hair samples of 15 mammal species: two fossil sloths, five modern xenarthran species under controlled feeding conditions (i.e., zoo specimens, Table 1), and eight wild omnivore species (Table 2, Dataset SI). Diet samples for the zoo specimens also were analyzed directly (Table S3). All amino acid compound specific (AACSIA) data for other mammals available in the literature (45 taxa) were also included, among these: 14 terrestrial primary consumers, 16 terrestrial secondary consumers, and 15 marine consumers. Statistical tests were two-tailed, and analyses were conducted with both parametric (two-sample t-test, ANOVA) and nonparametric (Mann–Whitney, Kruskal–Wallis) tests for significance. Groups do not have equal variances (Levene’s test *p* = 0.005), likely because the omnivore and marine consumers categories both include a broad range of diets (see main text), but differences between groups also were significant under an unequal variance t-test (Welch t test, Table S4).

### Sampling and analyses

All samples were analyzed for bulk and amino acid compound specific (AACSIA) δ^15^N at the Department of Earth Sciences, University of Hawaii at Manoa. To evaluate the temporal homogeneity (or heterogeneity) of the isotopic signal recorded in the modern sloth with a monospecific diet (i.e., *Bradypus*) and in the fossil species (*Mylodon* and *Nothrotheriops*) with unknown diets, hair strands for these three species were sectioned in 0.5 or 1 cm and analyzed for bulk δ^15^N prior to AACSIA (Fig. 2, Table S7). If significant differences were found among the bulk δ^15^N of the different hair sections, these were analyzed separately for AACSIA δ^15^N; otherwise, sections were homogenized. For the fossil samples, hair strands of one individual per species were sectioned in 1 cm increments and analyzed for δ^15^N and molar C:N ratio (Fig. S1, Table S7). For all the zoo species (except *Cyclopes*), the proximal 1 cm (expected to more closely record the diets sampled than distal sections, as this represents the most recently formed part of the hair) were homogenized and analyzed for δ^15^N (bulk and for AACSIA). For *Cyclopes,* possessing very thin and short hair, entire hair strands were homogenized for both bulk and AACSIA δ^15^N. Dietary items for the species with mixed diets were isolated and analyzed for bulk δ^15^N. Mixed diets were homogenized in the proportion of each individual dietary item given to the individual animals at the zoo and then analyzed for δ^15^N AACSIA. Variability of the isotopic values for the two modern sloths’ diets in the zoo was accounted for by analyzing samples on a weekly, monthly, and yearly basis (Tables S8–S10).

### Bulk δ^15^N values of hair keratin and C:N ratios for the fossil species

The δ^15^N value of the proximal first centimeter was higher than the remaining length of the hair for both species, so this first centimeter was conservatively excluded for AACSIA (Fig. S1, Table S7). Significant differences (*p*-value < 0.01) were found between *Mylodon darwinii*’s hair segments 3–4 plus 7–10 (mean = 2‰ ± 0.1), and segments 2 plus 5–6 (mean = 3.5‰ ± 0.6). Therefore, we homogenized segments 3–4 plus 7–10, and 2 plus 5–6, and analyzed those for AACSIA δ^15^N (Fig. S1). In contrast, after excluding the first centimeter (δ^15^N = 17.5‰), the δ^15^N values of the remaining hair segments of *Nothrotheriops* are extremely consistent and do not differ significantly from each other (mean = 16‰ ± 0.1, Fig. 2).

### Nitrogen isotope analyses of amino acids

Amino acids were chemically isolated and purified from sample material prior to derivatization and esterification in preparation for GC-IRMS analysis, as described in^56^. Samples were then analyzed using a Thermo Scientific Delta V Plus mass spectrometer interfaced with a Thermo Finnigan Trace GC gas chromatograph via a Thermo Finnigan GC-C III. Amino acids measured using this technique include alanine (Ala), glycine (Gly), isoleucine (Iso), leucine (Leu), lysine (Lys), methionine (Met), phenylalanine (Phe), proline (Pro), serine (Ser), threonine (Thr), tyrosine (Tyr), and valine (Val). Additionally, the terminal amide groups in glutamine (Gln) and aspartamine (Asn) are cleaved during the chemical isolation of amino acids, the result being the conversion of these amino acids to glutamic acid (Glu) and aspartic acid (Asp), respectively. Thus, we measured the isotope values of combined Glu + Gln (termed Glx) and Asn + Asp (termed Asx). Internal reference compounds, L-2-Aminoadipic acid (AAA) and L-(+)-Norleucine (Nor) of known nitrogen isotopic composition, were co-injected with samples and used to determine accuracy and precision. Between triplicate runs of each sample, a suite of these 14 amino acids of known isotopic composition were analyzed; AAA and Nor were also co-injected with these amino acids. For isotopic correction of unknown amino acids, a linear correction is derived from the amino acid suites run immediately before and after the triplicate sample analysis and applied to measured isotope ratios. All amino acids were analyzed in triplicate and isotopic values are reported in δ-notation relative to atmospheric N_2_.

### Ethics

The protocols used to collect hair samples from museum/zoo specimens conform to the legal requirements and institutional guidelines of the Peruvian and US Governments (permit # 003150-SERFOR, PE1620, PE1621, USFWS NY2039715). All applicable international, national, and/or institutional sampling methods were carried out in accordance with relevant guidelines and regulations. When appropriate, this research adheres to the ARRIVE guidelines.

## Supplementary Information

Supplementary Information 1.

Supplementary Information 2.
