# Supplementary material for: Isotope data from amino acids indicate Darwin’s ground sloth was not an herbivore
Source: Sci Rep. 2021 Oct 7;11:18944. doi: 10.1038/s41598-021-97996-9 (PMC8494799; doi:10.1038/s41598-021-97996-9)
Supplement: Supplementary file 2 — Supplementary Information 2. [file 41598_2021_97996_MOESM2_ESM.docx]

**SUPPLEMENTARY INFORMATION FOR**

**ISOTOPE DATA FROM AMINO ACIDS INDICATE DARWIN’S GROUND SLOTH WAS NOT AN HERBIVORE**

Julia V. Tejada*, John J. Flynn, Ross MacPhee, Tamsin O’Connell, Thure E. Cerling, Lizette Bermudez, Carmen Capuñay, Natalie Wallsgrove, Brian N. Popp

*Corresponding author

Email: [julia.tejada@umontpellier.fr](mailto:julia.tejada@umontpellier.fr)

**This file includes:**

Supplementary text

Figures S1

Tables S1 to S10

**Other supplementary materials for this manuscript include the following:**

Datasets S1

**Supplementary Information Text**

**New animal and diet samples analyzed in this study**

**Fossil samples.** *Mylodon darwinii* (Mylodon Cave, Chile, 2 specimens, 3 samples, AMNH FM 96263) and *Nothrotheriops shastensis* (New Mexico, USA, 1 specimen, YPM VP 013198).

**Zoo specimens, with controlled-feeding dietary data**. Five modern xenarthran species from the Huachipa Zoo (Lima, Peru): *Bradypus variegatus* (three-toed sloth; 2 specimens, 18 samples), *Choloepus hofffmanii* (two-toed sloth; 5 specimens, 9 samples), *Cyclopes didactylus* (silky anteater; 2 specimens, 3 samples), *Tamandua tetradactyla* (lesser anteater, 4 specimens, 5 samples), and *Myrmecophaga tridactyla* (giant anteater; 2 specimens, 2 samples). The three-toed sloth *Bradypus* is an obligate herbivore in the wild and at the zoo, where it is fed a monospecific diet exclusively composed on young sprouts of *Ficus elastica*, the rubber plant. In contrast, the two-toed sloth *Choloepus* was fed a combination of 8 items at the zoo, including one component of animal origin. The zoo diets of the three anteater species, obligately insectivorous in the wild, include elements that are artificially enriched in specific amino acids. The silky or pygmy anteater *Cyclopes didactylus* was fed a 1:1 formula of two animal protein mixtures (milk replacers), and the anteaters *Myrmecophaga* and *Tamandua* both were fed a combination of 9 items, including at least two of animal origin. Analytical details for all of the hair and diet samples are described below.

**Modern wild omnivores** (all samples from the AMNH Mammalogy collections). *Martes americana* (AMNH 12771, American marten, Loc: Canada, New Brunswick), *Nasua nasua* (AMNH 95095, South American coati, Loc: Brazil, Para), *Urocyon cinereoargenteus* (AMNH 243446, common gray fox, Loc: USA, Florida), *Genetta genetta* (AMNH 187724, common genet, Loc: Kenya, Garissa), *Chrysocyon jubatus* (AMNH 41315, maned wolf, Loc: Brazil, Mato Grosso), *Chaetophractus vellerosus* (AMNH 40067, screaming hairy armadillo, Loc: Argentina, Mendoza), *Atelerix frontalis* (AMNH 207247, southern African hedgehog, Loc: Zimbabwe, Matabeleland North), and *Saimiri boliviensis* (AMNH 211611, Bolivian squirrel monkey, Loc: Bolivia, Beni Mamore).

**Amino acid δ^15^N analyses from hair keratin and dietary samples**. Thirteen amino acids were consistently measured in all samples: four “source” amino acids (phenylalanine [Phe], glycine [Gly], lysine [Lys], and serine [Ser]), eight “trophic” amino acids (glutamic acid [Glx], proline [Pro], alanine [Ala], aspartic acid [Asx], valine [Val], leucine [Leu], and isoleucine [Iso]), and the “metabolic” amino acid threonine (Thr). Nomenclature for “source”, “trophic”, and “metabolic” amino acids follows ^17,19^. In some samples, δ^15^N values for the source amino acids tyrosine (Tyr) and methionine (Met) also were recovered. The metabolic AA Thr is the most ^15^N depleted in all samples analyzed (other than for *Bradypus*’ diet, *Ficus elastica*, the rubber plant), so when describing the lower end of the δ^15^N range of AA values, in addition to Thr we also include the AA with the next lowest δ^15^N value. The analysis of both consumer tissue and diet samples of the zoo specimens permitted calculation of the offset in δ^15^N values from dietary protein to hair keratin for each AA. This is referred to as “big delta” (Δ^15^N_consumer-diet_); see also the main text.

**Supplementary results**

**Bulk δ^15^N of hair keratin and diets of zoo species**

***Bradypus variegatus* (three-toed sloth).** Hair strands of two *Bradypus* specimens (sampled in 2018 and 2019) were sectioned in 4 or 5 segments of 0.5 cm each (Table S7). The median bulk δ^15^N of *Bradypus*’ hair samples is 10.9‰ ± 0.3‰, ranging from 10.5‰ to 11.4‰ (Fig. S1, Table S7). Differences in bulk δ^15^N for *Bradypus*’ hair segments within and among specimens are significant (*p*-value= 0.02 and 0.05 respectively), but within instrument error (0.4-0.6‰). The median δ^15^N of *Bradypus*’ diet was 7.1‰ ± 0.7‰ (Table S8).

***Choloepus hoffmanii* (two-toed sloth).** The median bulk δ^15^N of the proximal 1 cm of the 11 specimens analyzed was 7.2‰ ± 1.1‰, ranging from 6.7‰ to 8‰ (Fig. S1, Table S9). The median δ^15^N of the homogenized diet of *Choloepus* was 2.5‰ (± 0.2‰), ranging from 2‰ to 2.8‰ (Table S9).

***Cyclopes didactylus* (pygmy anteater).** The median δ^15^N of the three *Cyclopes* specimens was 8.7‰ ± 0.2‰ (Fig. 2, Table S10). *Cyclopes’* diet at the zoo is a two-component animal protein milk replacer mixture (whey^TM^ protein + Esbilac® powder [dietary supplement]). The average δ^15^N for the zoo diet of this species was 5.3‰ ± 0.1‰ (Table S10).

***Tamandua tetradactyla* (lesser anteater) and *Myrmecophaga tridactyla* (giant anteater).** The median δ^15^N (proximal 1 cm) of *Tamandua*’s hair is 5.9‰ ± 0.2‰ and that of *Myrmecophaga* is 7‰ ± 0.2‰ (Table S10). *Tamandua* and *Myrmecophaga*’s mixed diet (both are fed the same diet at the zoo) showed a δ^15^N of 5.3‰ (Table S10).

**AACSIA δ^15^N of hair keratin and diet samples**

The C:N molar ratios obtained for both fossil sloths, *Mylodon* (3.6) and *Nothrotheriops* (3.1), are within the range expected for extant mammal hair samples (i.e., <4) indicating that these fossil samples are well preserved (Table S7). The AAs with highest δ^15^N value in hair keratin for all mammalian species were proline, valine, and glutamic acid (Table 1-2, Table S1). The “metabolic” amino acid threonine was the most depleted in ^15^N for all samples.

**Modern sloths.** Median values of hair keratin for both modern sloths *Bradypus variegatus* and *Choloepus hoffmanni* are bracketed by threonine and lysine at the lower end, and proline and glutamic acid at the upper end (Table 1). Individual AA δ^15^N values of *Ficus elastica* (the rubber plant, *Bradypus*’ diet) range from 6‰ (Leu) to 18.6‰ (Phe) while dietary AA δ^15^N values in *Choloepus* are bracketed by Thr and Ser at the lower end (0.3‰ and 1.2‰ respectively) and Pro and Ala at the upper end (8.6‰, Table S2). There are no significant differences between the mean δ^15^N of source and trophic AAs (p-value>0.2) in the hair keratin of either sloth species, nor between source and trophic AAs of their diets (p-value>0.1). Of the four source AAs evaluated, only Lys and Gly have Δ^15^N_consumer-diet_ within one standard deviation of ~0‰ in *Bradypus*, while Lys is the only source AA with a Δ^15^N_consumer-diet_ value close to 0‰ in *Choloepus*. In *Bradypus,* offsets in δ^15^N from diet to hair keratin (Δ^15^N_consumer-diet_) vary among AAs, ranging from Thr and Phe (-8.5‰ and -4.5‰ respectively) to Pro and Iso (~6‰) (Table S2). Differences in mean Δ^15^N_consumer-diet_ between source (2.6‰) and trophic (3.9‰) AAs of *Bradypus*’ hair keratin are not significant (p-value=0.5).

**Zoo fed (captive) anteaters***.* AA δ^15^N of hair keratin in the three anteater species (*Cyclopes didactylus, Myrmecophaga tridactyla,* and *Tamandua tetradactyla*) is bracketed by Thr, Tyr, and Gly at the lower end, and Iso (*Cyclopes*) and Pro (*Myrmecophaga* and *Tamandua*) at the upper end (Table S1). There are no significant differences between the mean δ^15^N of source (8.2‰) and trophic (12.8‰) AAs (p-value=0.08) in *Cyclopes* hair keratin. In contrast, differences between the mean δ^15^N of source (4.5‰) and trophic (10.1‰) AAs of *Tamandua* and *Myrmecophaga* hair keratin are significant (p-value<0.01). Differences in the mean Δ^15^N_consumer-diet_ between source and trophic AAs are not significant (p-value>0.05, Table S2). All Δ^15^N_consumer-diet_ values of source AAs differ significantly from 0‰ in *Cyclopes*. Among source AAs of *Tamandua* and *Myrmecophaga*, Ser and Lys have Δ^15^N_consumer-diet_ values within one standard deviation of ~0‰. AA δ^15^N of hair keratin range from lows in Thr and Tyr for both *Myrmecophaga* (-13‰ and -2.3‰ respectively) and *Tamandua* (-12‰ and 1.8‰ respectively) to a high in Pro (15.3‰ in *Myrmecophaga* and 14.4‰ in *Tamandua*)*.* The δ^15^N of dietary AAs (the same for both species) range from lows in Thr and Lys (-1.1‰ and 2.8‰) to a high in Ala (11.6‰, Table S1). Differences between the mean δ^15^N of source (4.5‰) and trophic (10.1‰) AAs of *Tamandua* and *Myrmecophaga* hair keratin are significant (p-value<0.01). However, the δ^15^N of dietary source (7.2‰) and trophic (8.6‰) AAs do not differ significantly (p-value=0.5). Differences in the mean Δ^15^N_consumer-diet_ between source (-1.4‰) and trophic (1.5‰) AAs also are not significant (p-value=0.06). Among source AAs, Ser and Lys have Δ^15^N_consumer-diet_ values within one standard deviation of ~0‰.

**Fossil sloths.** Differences in the δ^15^N values of individual AAs between the two fossil sloth species (*Mylodon darwinii* and *Nothrotheriops shastensis*) are significant (p-value<0.001). The three samples of *Mylodon* show consistent patterns of δ^15^N AACSIA (Table 1, Fig. 2), with median values ranging from -7.2‰ and -3.4‰ (Thr and Tyr respectively) to 5.9‰ (Iso and Pro). The largest intraspecific variation in δ^15^N is observed in Ala, with an almost 5‰ difference between the two individuals of *Mylodon* sampled. There are intraspecific differences (p-value<0.001) among source (mean δ^15^N= 0.8‰) and trophic AAs (mean δ^15^N= 4.9‰). *Nothrotheriops* AA δ^15^N values vary widely (15‰), ranging from 4.5‰ and 9.8‰ (Thr and Lys respectively) to 19.6‰ (Glx). The AA values of *Nothrotheriops* are the most enriched in ^15^N of all the species analyzed (see main text). Differences between the mean of source (14.1‰) and trophic (17.1‰) AAs of *Nothrotheriops* hair keratin are not significant (p-value= 0.1).

**New AACSIA δ^15^N data of modern wild omnivores**. Excluding Thr, AA δ^15^N values in all omnivore species sampled are bracketed by Lys and Pro at the lower and upper ends, respectively (Table 2). Source and trophic AAs in the eight modern omnivores evaluated differ significantly (all p-values <0.02). Of the eight species newly analyzed here, the American marten (*Martes americana*) showed the lowest δ^15^N values for most AAs. In contrast, the common genet *Genetta genetta* showed the highest δ^15^N values for all AAs, with values comparable to some marine consumers (Table 2).

**Testing the accuracy of the trophic position equation (TP Eq)**

We evaluated the predictive power of the widely applied trophic position equation (TP Eq) of ^20^ by calculating the trophic level of modern species with known trophic levels or controlled diets (Table S6). The equation for any studied tissue sampled is described as:

$$TP=\left( \frac{{\delta15N}_{Glx studied tissue}-{\delta15N}_{Phe studied tissue}-\beta}{TDF} \right)+1$$

β is the difference between the δ^15^N of Glx (δ^15^N_Glx_) and Phe (δ^15^N_Phe_) in the ecosystem’s primary producer, and TDF is the offset in δ^15^N_Glx_ relative to δ^15^N_Phe_ with increasing trophic level. The trophic levels estimated via this equation were then assessed for reliability relative to the known diets/trophic level of the animals sampled. A TP of 1 is expected for primary producers, TP=2 for primary consumers (i.e., herbivores), TP=3 for carnivores, TP=4 for hypercarnivores. Intermediate values have not been well defined before but are interpreted to represent intermediate trophic positions between the bracketing trophic levels, reflecting mixed diets.

The TP Eq of ^20^ treats β (-8.4‰ for C3, 0.4 for C4, and 3.4 for marine ecosystems) and TDF (7.6‰) as constants. This TP equation predicted the trophic position of *Bradypus* as an herbivore (average TP= 2.1), but failed to accurately reconstruct the trophic positions of the two-toed sloth *Choloepus* and the three anteaters. Indeed, the TPs obtained for the three specimens of *Choloepus* varied from 2.1 to 3.2 (SI Dataset), and thus individuals are interpretable as either a pure herbivore or a secondary consumer. *Cyclopes*, a species with a mixed diet in the zoo that includes animal whey protein (protein isolates from cow’s milk) showed a TP of 2.1, which would correspond to that expected for an herbivorous primary consumer. The trophic positions obtained for the anteaters *Tamandua* and *Myrmecophaga,* whose omnivorous, mixed diets at the zoo include horse meat and other elements of animal origin, varied from 2.0 for *Myrmecophaga* and one *Tamandua* specimen, to 2.7 for the second individual of *Tamandua*, reflecting trophic level interpretations as a primary consumer for the lower TP values to intermediate between primary and secondary consumer for the higher value (SI Dataset). The TP obtained for both fossil sloth species, *Mylodon* and *Nothrotheriops*, was consistent among individuals at 2.5. Even excluding the anteaters, whose artificial diets with mixed pant-animal components could be precluding a correct trophic placement, the TP equation also overestimated the trophic positions of 7 of the 8 modern wild omnivore species newly analyzed (Table S6). With the exception of the American marten (TP=2.4), TP values for the other omnivorous species ranged from 2.8 to 3.5 (median TP= 2.9) which would better fit a carnivory interpretation. Although interpretation of intermediate values derived from the TP Equation is not straightforward, a TP of 2.7 obtained from *Homo neanderthalensis* (Spy Cave, Belgium) was reconstructed as this species relying mostly on meat (~80% of the diet, ^23^), showing that mammals with TP values above 2.5 are usually interpreted as incorporating substantial animal-origin components in their diets. It is noteworthy that although TPs of some omnivores were overestimated, with values interpretable as reflecting obligate carnivory (e.g., TPs of *Genetta*= 3.5, *Chrysocyon*= 3.1, *Nasua*= 3), none of the primary consumers showed TP values indicating a higher trophic level (all of their TP values range from 1.8 to 2.2). The TP of most marine mammals were underestimated. For instance, the killer whale *Orcinus orca*, the bowhead *Balaena mysticetus*, or the fur seal *Arctocephalus pusillus* are reconstructed as primary consumers, while the sperm whale *Physeter macrocephalus* is reconstructed as an omnivore (Table S6). Concerning the fossil data, the three fossil sloth specimens were consistently reconstructed as omnivores, with TPs of 2.5, i.e., intermediate between an herbivore and a carnivore.

**
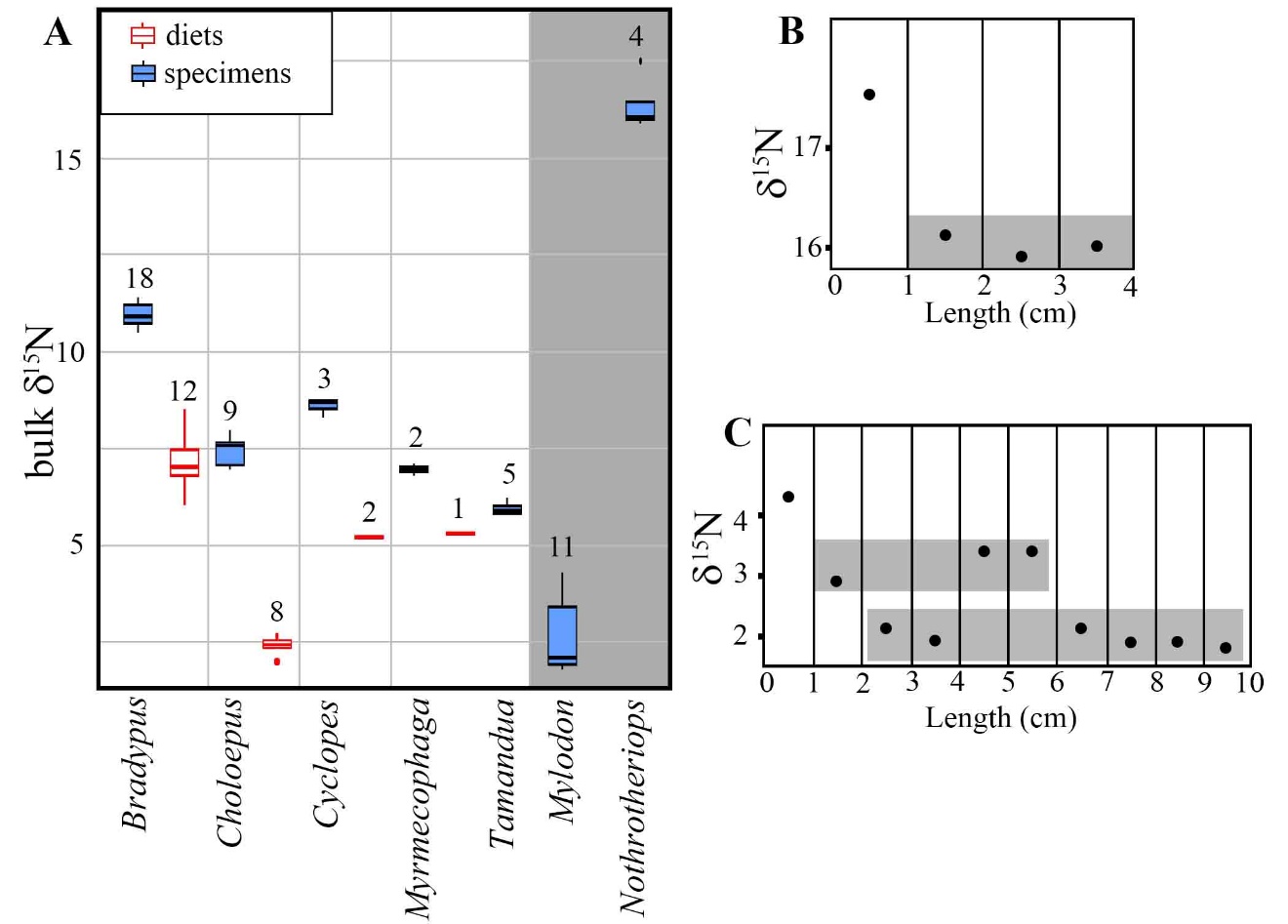
**

**Fig. S1.** Nitrogen isotope values of bulk hair and diet samples for the five xenarthran species with known feeding regimes in the zoo, and of bulk hair two fossil samples. A. Numbers above boxplots represent number of samples, *Myrmecophaga* and *Tamandua* placed together because they share the same diet at the zoo. B and C. Serial sampling of hair strands for one specimen per fossil species. Samples inside grey boxes in B and C were homogenized for δ^15^N AACSIA.

**Table S1.** AA and bulk tissue δ^15^N values for modern zoo-fed, omnivorous-diet anteaters. ∆ Glx-Phe is the offset between δ^15^N_Glx_ and δ^15^N_Phe_ values.

| **Sample** | **δ^15^N (‰)** | | | | | | | | | | | | | | ∆ Glx-Phe |
| --- | --- | --- | --- | --- | --- | --- | --- | --- | --- | --- | --- | --- | --- | --- | --- |
|  | **Ala** | **Gly** | **Thr** | **Ser** | **Val** | **Leu** | **Iso** | **Pro** | **Asx** | **Glx** | **Phe** | **Tyr** | **Lys** | **Bulk** |  |
| *Cyclopes didactylus* | 10.8 | 3.2 | -9 | 9.3 | 11.3 | 11.8 | 15.3 | 14.7 | 10.9 | 14.5 | 14.8 | 7.72 | 5.78 | 8.3 | -0.3 |
| *Tamandua tetradactyla* | 10.1 | 1.54 | -12 | 6 | 8.06 | 6.71 | 12.1 | 14.9 | 7.58 | 10.2 | 5.3 | 2.35 | 2.74 | 5.8 | 4.9 |
|  | 8.94 | 3.14 | -12 | 7.8 | 8.23 | 7.88 | 11.1 | 13.9 | 7.3 | 9.74 | 10.4 | 1.17 | 2.1 | 5.8 | -0.7 |
| Average *Tamandua* | **9.52** | **2.34** | **-12** | **6.9** | **8.14** | **7.29** | **11.6** | **14.4** | **7.44** | **9.95** | **7.86** | **1.76** | **2.42** |  | **2.1** |
| *Myrmecophaga tridactyla* | 10.5 | 3.71 | -13 | 8.7 | 8.25 | 8.81 | 10.6 | 15.3 | 9.1 | 11 | 12.1 | -2.3 | 1.96 | 7.1 | -1.1 |

**Table S2.** AA and bulk tissue δ^15^N values for dietary samples of the zoo specimens included in this study. Δ^15^N_consumer–diet_= δ^15^N_consumer_ – δ^15^N_diet_. δ^15^N_consumer_ from Table 1 and Table S1.

| **Sample** | **δ^15^N (‰)** | | | | | | | | | | | | | |
| --- | --- | --- | --- | --- | --- | --- | --- | --- | --- | --- | --- | --- | --- | --- |
|  | **Ala** | **Gly** | **Thr** | **Ser** | **Val** | **Leu** | **Iso** | **Pro** | **Asx** | **Glx** | **Phe** | **Tyr** | **Lys** | **Bulk** |
| *Bradypus’* diet (*Ficus elastica*) | 10.9 | 11.9 | 8.8 | 7 | 9.27 | 5.98 | 8.37 | 9.2 | 9.44 | 11 | 18.6 | - | 7.8 | 8.5 |
| ∆consumer-diet | **1.11** | **-0.6** | **-8.5** | **5.7** | **4** | **5.72** | **5.78** | **6.01** | **2.14** | **3.54** | **-4.5** | **-** | **1.9** |  |
| *Choloepus’* diet (mixed) | 8.55 | 6.82 | 0.3 | 1.2 | 4.37 | 2.39 | 5.08 | 8.58 | 7.08 | 5.28 | 4.53 | - | 2.2 | 2.4 |
| ∆consumer-diet | **0.69** | **-1.7** | **-5.9** | **8.1** | **4.61** | **5.71** | **5.71** | **2.96** | **1.5** | **6.21** | **3.64** | **-** | **0.2** |  |
| *Cyclopes’* diet (mixed of 2 items) | 7.03 | 6.23 | -0.3 | 5.3 | 8.09 | 3.98 | 7.26 | 5.7 | 6.97 | 7.57 | 8.57 | -1.2 | 1.1 | 5.2 |
| ∆consumer-diet | **3.76** | **-3** | **-8.7** | **4** | **3.19** | **7.83** | **8.05** | **8.98** | **3.9** | **6.9** | **6.24** | **8.94** | **4.7** |  |
| *Tamandua* & *Myrmecophaga’s* mixed diet | 11.6 | 9.69 | -1.1 | 6.2 | 6.61 | 5.68 | 8.63 | 9 | 9.13 | 9.49 | 9.86 | - | 2.8 | 5.3 |
| ∆consumer-diet  *Myrmecophaga* | **-1.1** | **-6** | **-12** | **2.5** | **1.64** | **3.13** | **1.96** | **6.31** | **-0** | **1.5** | **2.27** | **-** | **-0.9** |  |
| ∆consumer-diet  *Tamandua* | **-2.1** | **-7.3** | **-11** | **0.7** | **1.53** | **1.62** | **2.97** | **5.42** | **-1.7** | **0.47** | **-2** | **-** | **-0.4** |  |

**Table S3.** Summary of the β (δ^15^N_Glx_-δ^15^N_Phe_ in the diets), TDF, and ∆δ^15^N_Glx_-δ^15^N_Phe_ values for all captive, zoo-fed species with known diets in this study.

| **Species** | ***Bradypus variegatus*** | ***Choloepus hoffmanii*** | ***Cyclopes didactylus*** | ***Myrmecophaga tridactyla*** | ***Tamandua tetradactyla*** |
| --- | --- | --- | --- | --- | --- |
| **Diet** | *Ficus elastica* | mixed diet | mixed diet | mixed diet | mixed diet |
| **β value** | -7.61 | 0.75 | -1.0 | -0.37 | -0.37 |
| **Consumer** ∆**δ^15^N_Glx_**-**δ^15^N_Phe_** | 0.44 | 3.32 | -0.34 | -1.14 | 2.09 |
| **TDF** | 8.05 | 2.57 | 0.66 | -0.77 | 2.46 |

**Table S4.** Δδ^15^N_Glx_-δ^15^N_Phe_ per dietary category. Summary statistics (A), one-way ANOVA (B), Tukey’s pairwise comparison, pairwise comparisons and Welch two sample t-test (C), and ANCOVA. Kruskal-Wallis test for medians (5.34E-08) indicate that differences in ∆δ^15^N_Glx_-δ^15^N_Phe_ among dietary categories are significant. The omnivores category excludes the zoological anteaters (see main text and SI for explanation). Df= degrees of freedom, F= F-statistics, Pr>F= p-value associated with the F statistics.

| **A.** | **Herbivores** | | **Marine consumers** | **Omnivores** | ***Mylodon*** | ***Nothrotheriops*** |
| --- | --- | --- | --- | --- | --- | --- |
| N | 15 | | 15 | 18 | 3 | 1 |
| Min | -2.3‰ | | 12.1‰ | 1.2‰ | 2.6‰ | 1.9‰ |
| Max | 0.4‰ | | 23.7‰ | 10.7‰ | 3.1‰ |  |
| Mean | -0.8‰ | | 15.9‰ | 5.0‰ | 2.8‰ |  |
| Median | -0.7‰ | | 14.7‰ | 5.2‰ | 2.7‰ |  |
| Variance | 0.64‰ | | 12.02‰ | 5.06‰ |  |  |
| Std. Error | 0.2‰ | | 0.9‰ | 0.6‰ |  |  |
| Std. Dev | 0.8‰ | | 3.5‰ | 2.7‰ | 0.3‰ |  |
| Shapiro-Wilk (*p*) | 0.58 | | 0.03 | 0.46 |  |  |
|  | | | | | | |
| **B.** | **Sum of sqrs** | | **df** | **Mean square** | **F** | ***p*** |
| Between groups | 2170.3 | | 2 | 1085.1 | 185.4 | <0.0001 |
| Within groups | 263.4 | | 45 | 5.9 |  |  |
| Total | 2433.7 | | 47 |  |  |  |
| **C.** | | | **Tukey’s pairwise, Mann-Whitney, Kruskal-Wallis** | **Welch two-sample t-test** | | |
|  |  |  |  | **t-value** | **p-value** | **Difference btw means** |
| Herbivore vs Omnivore | | | 0.0001 | t(21.9)= -10.2 | 8.348e-10 | 5.82 |
| Herbivore vs Marine | | | 0.0001 | t(15.5)= -18.2 | 7.127e-12 | 16.72 |
| Marine vs Omnivore | | | 0.0001 | t(23.2)= -10.5 | 2.849e-10 | 10.91 |
| **D. ANCOVA** | |  | | | | |
| **δ^15^N_Glx_~δ^15^N_Phe_ *diet** | | **df** | **Sum Sq** | **Mean Sq** | **F** | **Pr(>F)** |
| δ^15^N_Phe_ | | 1 | 28.4 | 28.4 | 4.77 | 0.0345 |
| Diet | | 3 | 1998 | 666 | 111.7 | <2e-16 |
| δ^15^N_Phe_:diet | | 3 | 6.5 | 2.2 | 0.36 | 0.78 |
| Residuals | | 43 | 256.5 | 6 |  |  |
|  | | | | | | |
| **δ^15^N_Glx_~δ^15^N_Phe_+diet** | | **df** | **Sum Sq** | **Mean Sq** | **F** | **Pr(>F)** |
| δ^15^N_Phe_ | | 1 | 28.4 | 28.4 | 4.98 | 0.03 |
| Diet | | 3 | 1998.2 | 666.1 | 116.51 | <2e-16 |
| Residuals | | 46 | 263 | 5.7 |  |  |

**Table S5.** Correlation values (in Fig. 1 of the main text) for δ^15^N_Glx_ and δ^15^N_Phe_ and ∆δ^15^N_Glx_-δ^15^N_Phe_.

|  | **N** | **R^2^** | **p-value** | **Intercept** | **Slope** | **Median ∆δ^15^N_Glx_-δ^15^N_Phe_** | **Mean ∆δ^15^N_Glx_-δ^15^N_Phe_** | **Range of ∆δ^15^N_Glx_-δ^15^N_Phe_** |
| --- | --- | --- | --- | --- | --- | --- | --- | --- |
| Herbivores | 15 | 0.96 | 2.27e-10 | -2.54 | 1.16 | -0.7 | -0.7 | -2.3 to 0.4 |
| Modern omnivores (all, including zoo specimens) | 21 | 0.13 | 0.11 | 9.55 | 0.43 | 4.6 | 4.32 | -1.1 to 10.7 |
| Modern omnivores (excluding zoo anteaters because of artificial diets) | 18 | 0.28 | 0.03 | 6.83 | 0.79 | 5.2 | 5.0 | 1.2 to 10.7 |
| Marine consumers | 15 | 0.52 | 0.002 | 14.44 | 1.17 | 13.7 | 15.46 | 11.1 to 23.7 |
| *Mylodon* | 3 | - | - | - | - | 2.7 | 2.8 | 2.6 to 3.1 |
| *Nothrotheriops* | 1 | - | - | - | - | 1.9 | | - |
| Herbivores + fossil sloths | 19 | 0.86 | 9.367e-09 | 1.08 | 0.88 |  |  |  |
| Herbivores + *Nothrotheriops* only | 16 | 0.96 | 1.316e-11 | -3.22 | 1.23 |  |  |  |
| Omnivores + fossil sloths | 22 | 0.68 | 2.579e-06 | 4.72 | 0.98 |  |  |  |
| Omnivores + *Mylodon* (excluding outliers *Nothrotheriops* and *Genetta*) | 20 | 0.71 | 3.102e-06 | 3.97 | 1.05 |  |  |  |

**Table S6.** Summary data from this study and literature compilation for mammals with trophic position (TP_Eq) estimations (following ^20^) with constant β [8.4‰ for C3 and -3.4‰ for marine ecosystems] and TDF [7.6‰] values. Note that TP reconstructions for marine consumers using that TP equation (^20^) are particularly underestimated. Fossil sloths in bold.

**Table S7.** A. Bulk δ^15^N of the hair samples for the two specimens of *Bradypus variegatus* sampled in 2018 and 2019. Zoo ID refers to the identification (chip) number of the specimen at the zoo. B. Bulk δ^15^N (‰ vs. AIR) and C:N ratio (mol/mol) for hair segments of the fossil sloths *Mylodon darwinii* and *Nothrotheriops shastensis*.

| **A.** | | **Bulk δ^15^N (‰) *Bradypus variegatus*** | | | | | | | | | | |
| --- | --- | --- | --- | --- | --- | --- | --- | --- | --- | --- | --- | --- |
| **Hair segments** | | | **Zoo ID: _372836** | | | | | **Hair segments** | | | **Zoo ID: _372840** | |
|  |  |  | **2018** | | | **2019** | |  |  |  | **2018** | **2019** |
| 0 – 0.5 cm | | | 11.2 | | | 10.8 | | 0 – 0.5 cm | | | 11.1 | 11 |
| 0.5 – 1 cm | | | 10.9 | | | 10.7 | | 0.5 – 1 cm | | | 11.1 | 10.7 |
| 1 – 1.5 cm | | | 11.2 | | | 10.5 | | 1 – 1.5 cm | | | 11.2 | 10.7 |
| 1.5 – 2 cm | | | 11.4 | | | 10.5 | | 1.5 – end | | | 10.8 | 10.6 |
| 2 - end | | | 11.3 | | | NA | |  | | |  |  |
| **Median** | | | 11.2 | | | 10.6 | | **Median** | | | 11.1 | 10.7 |
| **B.** | | **Bulk δ^15^N (‰) of the fossil sloth species** | | | | | | | | |  |  |
| ***Mylodon darwinii*** | | | | | | ***Nothrotheriops shastensis*** | | | | |  |  |
| **Segments (cm)** | | | | **δ^15^N** | **C:N** | **Segments (cm)** | | | **δ^15^N** | **C:N** |  |  |
| 1 | 0-1 | | | 4.3 | 3.5 | 1 | 0-1 | | 17.5 | 3.1 |  |  |
| 2 | 1-2 | | | 2.9 | 3.6 | 2 | 1-2 | | 16.1 | 3.1 |  |  |
| 3 | 2-3 | | | 2.1 | 3.5 | 3 | 2-3 | | 15.9 | 3.1 |  |  |
| 4 | 3-4 | | | 1.9 | 3.6 | 4 | 3-4 | | 16.0 | 3.1 |  |  |
| 5 | 4-5 | | | 3.4 | 3.6 |  | | | | |  |  |
| 6 | 5-6 | | | 3.4 | 3.6 |  |  |  |  |  |  |  |
| 7 | 6-7 | | | 2.1 | 3.6 |  |  |  |  |  |  |  |
| 8 | 7-8 | | | 1.9 | 3.5 |  |  |  |  |  |  |  |
| 9 | 8-9 | | | 1.9 | 3.5 |  |  |  |  |  |  |  |
| 10 | 9-10 | | | 1.8 | 3.5 |  |  |  |  |  |  |  |

**Table S8**. Bulk δ^15^N of the *Bradypus*’s diet samples (*Ficus elastica*, rubber plant), sampled in 2016, 2018, and 2019 (ID numbers ending in -16, 2018, or 2019, respectively).

| ***Bradypus*’ diet** | **Sample ID** | **δ^15^N (‰)** |
| --- | --- | --- |
| *Ficus elastica* | BRA-DIET2019 | 8.5 |
|  | BRA-DIET2018 | 7.0 |
|  | BRA-05-01-16 | 6.1 |
|  | BRA-06-01-16 | 7.2 |
|  | BRA-07-01-16 | 6.0 |
|  | BRA-08-01-16 | 6.8 |
|  | BRA-09-01-16 | 7.4 |
|  | BRA-10-01-16 | 6.7 |
|  | BRA-11-01-16 | 6.9 |
|  | CHO-04-01-16H | 7.0 |
|  | BRA-05-01-16 | 7.7 |
|  | BRA-05-01-16 | 7.7 |
|  | **Median** | 7.1 ± 0.7 |

**Table S9**. A. Bulk δ^15^N of the hair samples for the different specimens of *Choloepus hoffmanii* sampled in 2018 (Cho-01-19 to Cho-05-19) and 2019 (Cho-08-19 to Cho-11-19). Zoo ID refers to the identification (chip) number of the specimen at the zoo. B. Bulk δ^15^N of *Choloepus*’ homogenized diet samples (ID numbers ending in -16 were sampled in 2016 and Di-2019 in that year).

| **A.** | **Bulk δ^15^N (‰) *Choloepus hoffmanii*** | | | | |
| --- | --- | --- | --- | --- | --- |
| **Zoo ID** | **Sample ID** | | | **2018** | **2019** |
| 373213 (Candy) | Cho-01-19, Cho-11-19 | | | 8.0 | 7.6 |
| 373191 (Beny) | Cho-02-19, Cho-10-19 | | | 7.7 | 7.6 |
| 373486 (Sandra) | Cho-03-19, Cho-09-19 | | | 6.9 | 6.7 |
| 372841 (Speedy) | Cho-05-19, Cho-08-19 | | | 7.2 | 7.1 |
| 373220 (Lia) | Cho-04-19 | | | 7.6 | - |
| **Median** | | | | 7.2 ± 1.1 | |
|  | | | |  | |
| **B.** | | **Bulk δ^15^N (‰) *Choloepus*’ diet** | | | |
| ***Choloepus*’ diet** | | | **Sample ID** | **δ^15^N (‰)** | |
| Sweet potato, carrot, broccoli, spinach, boiled quinoa, sprouts of rubber plant, Purina® Dog Chow | | | Cho-Di-2019 | 2.4 | |
|  |  |  | Cho-05-16 | 2.0 | |
|  |  |  | Cho-06-16 | 2.5 | |
|  |  |  | Cho-07-16 | 2.4 | |
|  |  |  | Cho-08-16 | 2.8 | |
|  |  |  | Cho-09-16 | 2.4 | |
|  |  |  | Cho-10-16 | 2.6 | |
|  |  |  | Cho-11-16 | 2.6 | |
|  |  |  | **Median** | 2.5 ± 0.2 | |

**Table S10**. A. Bulk δ^15^N of hair and diet samples of *Cyclopes didactylus*. B. Bulk δ^15^N of the hair and diet samples of *Myrmecophaga tridactyla* and *Tamandua tetradactyla* (both anteater species have the same diet at the zoo).

| 1. ***Cyclopes didactylus*** | | | | | | | | |
| --- | --- | --- | --- | --- | --- | --- | --- | --- |
| **Hair keratin** | | | |  | **Diet samples** | | | |
| **Sample ID** | | **δ^15^N (‰)** | |  | ***Cyclopes*’ diet** | **Sample ID** | | **δ^15^N (‰)** |
| Cy-01-19 (Paulina) | | 8.3 | |  | Esbilac® powder, whey protein (Gold standard 100% whey^TM^ French vanilla flavor) | Cy-Fre-Di19 | | 5.3 |
| Cy-02-19 (Freddy) | | 8.7 | |  |  | Cy-Pa-Di19 | | 5.2 |
| Cy-03-19 | | 8.7 | |  |  |  |  |  |
| Median | | 8.7 ± 0.2 | |  |  |  |  |  |
| 1. ***Myrmecophaga tridactyla* and *Tamandua tetradacyla*** | | | | | | | | |
| **Hair keratin** | | | |  | **Diet δ^15^N (‰)** | | | |
| **Taxon** | **Sample ID** | | **δ^15^N (‰)** |  | Purina® Cat Chow, soy milk, boiled egg, carrot, horse meat, banana, papaya, Aminomix® (protein supplement), Pecutrin® (vitamin and mineral supplement) | | 5.3 | |
| *Myrmecophaga* | Myr-01-19 | | 6.8 |  |  |  |  |  |
| *Myrmecophaga* | Myr-02-19 | | 7.1 |  |  |  |  |  |
| *Tamandua* | Ta-01-19 | | 5.8 |  |  |  |  |  |
| *Tamandua* | Ta-03-19 | | 5.9 |  |  |  |  |  |
| *Tamandua* | Ta-04-19 | | 5.8 |  |  |  |  |  |
| *Tamandua* | Ta-05-19 | | 6.0 |  |  |  |  |  |
| *Tamandua* | Ta-05-19 | | 6.2 |  |  |  |  |  |
